# An Innovative Therapeutic Option for the Treatment of Skeletal Sarcomas: Elimination of Osteo- and Ewing’s Sarcoma Cells Using Physical Gas Plasma

**DOI:** 10.3390/ijms21124460

**Published:** 2020-06-23

**Authors:** Josephine M. Jacoby, Silas Strakeljahn, Andreas Nitsch, Sander Bekeschus, Peter Hinz, Alexander Mustea, Axel Ekkernkamp, Mladen V. Tzvetkov, Lyubomir Haralambiev, Matthias B. Stope

**Affiliations:** 1 Department of Trauma, Reconstructive Surgery and Rehabilitation Medicine, University Medicine Greifswald, Ferdinand-Sauerbruch-Straße, 17475 Greifswald, Germany; josephinemarie.jacoby@stud.uni-greifswald.de (J.M.J.); ss166320@uni-greifswald.de (S.S.); an124100@uni-greifswald.de (A.N.); peter.hinz@med.uni-greifswald.de (P.H.); ekkernkamp@ukb.de (A.E.); 2 ZIK Plasmatis, Leibniz Institute for Plasma Science and Technology (INP), Felix-Hausdorff-Straße 2, 17489 Greifswald, Germany; sander.bekeschus@inp-greifswald.de; 3 Department of Gynecology and Gynecological Oncology, University Hospital Bonn, Venusberg-Campus 1, 53127 Bonn, Germany; alexander.mustea@ukbonn.de (A.M.); matthias.stope@ukbonn.de (M.B.S.); 4 Department of Trauma and Orthopaedic Surgery, BG Klinikum Unfallkrankenhaus Berlin Warener Straße 7, 12683 Berlin, Germany; 5 Department of Clinical Pharmacology, University Medicine Greifswald, 17489 Greifswald, Germany; mladen.tzvetkov@med.uni-greifswald.de

**Keywords:** osteosarcoma, Ewing’s sarcoma, cold atmospheric plasma, growth inhibitory effect, apoptosis, membrane integrity

## Abstract

Osteosarcoma and Ewing’s sarcoma are the most common malignant bone tumors. Conventional therapies such as polychemotherapy, local surgery, and radiotherapy improve the clinical outcome for patients. However, they are accompanied by acute and chronic side effects that affect the quality of life of patients, motivating novel research lines on therapeutic options for the treatment of sarcomas. Previous experimental work with physical plasma operated at body temperature (cold atmospheric plasma, CAP) demonstrated anti-oncogenic effects on different cancer cell types. This study investigated the anti-cancer effect of CAP on two bone sarcoma entities, osteosarcoma and Ewing’s sarcoma, which were represented by four cell lines (U2-OS, MNNG/HOS, A673, and RD-ES). A time-dependent anti-proliferative effect of CAP on all cell lines was observed. CAP-induced alterations in cell membrane functionality were detected by performing a fluorescein diacetate (FDA) release assay and an ATP release assay. Additionally, modifications of the cell membrane and modifications in the actin cytoskeleton composition were examined using fluorescence microscopy monitoring dextran-uptake assay and G-/F-actin distribution. Furthermore, the CAP-induced induction of apoptosis was determined by TUNEL and active caspases assays. The observations suggest that a single CAP treatment of bone sarcoma cells may have significant anti-oncogenic effects and thus may be a promising extension to existing applications.

## 1. Introduction

The most common primary solid malignancy of bone is osteosarcoma (OS). It is characterized by the production of osteoid by malignant mesenchymal cells [1,2]. In the general population, the annual incidence of OS is 4.4 per million and reaches its peak in the second decade of life [3,4]. Ewing’s sarcoma (ES) is the second most common malignant bone tumor of childhood and adolescence, with an incidence of 2.9 per million [4,5]. The most common primary localization of the OS is the distal femur, the proximal tibia, and the proximal humerus, with 50% originating in the knee [3,6]. In the axial skeleton with 10%, OS develops most frequently in the pelvis. In contrast, ES occurs primarily in the axillar skeleton, such as the pelvis (30%) and thorax (20%) [7].

OS and ES are highly aggressive types of cancer. Survival in patients with standard-risk and localized manifestation is between 55–75% for OS and 70–80% for ES [1,8,9]. Treatment includes local surgery, radiotherapy, and polychemotherapy, which are accompanied by acute and chronic side effects that can affect the quality of life of patients [1,10]. The five-year overall survival rate for OS and ES patients is about 63%. The ten-year survival rate for OS patients is 60.2%. For ES patients, it is 54.5%. Tumor response to chemotherapy, surgical treatment with adequate resection margins, and the development of postoperative metastases are the prognostic factors that significantly impact the overall survival rate of patients [11]. However, current regimens are characterized by acute and long-term toxicities that cause significant health problems and shorten patient life expectancy [12,13].

Consequently, new strategies for the treatment of OS and ES are required. In this context, the antitumor effects of cold physical plasma (cold atmospheric pressure plasma, CAP) have recently been suggested as novel anticancer therapy [14]. CAP is a highly energized gas at body temperature composed of numerous biologically active factors. For medical applications, CAP has the advantage of working at atmospheric pressure and tissue tolerable temperature. The CAP jet (kINPen med) used in this study generates the plasma by applying a high-frequency alternating voltage to a gas (argon). The argon ions remain slower due to their severity in the electric field, which prevents their acceleration and they therefore remain cold [15]. Plasma is generated in this effluent region and interacts with ambient air to form radical species (e.g., hydroxyl radical or non-radical (e.g., hydrogen peroxide) which subsequently interfere with cellular biomolecules [15]. UV-radiation, electric current and electromagnetic fields, however, play a significant role as well. [16,17,18]. When using the device as intended, this UV radiation is harmless to humans [19,20]. The antiseptic properties of CAP are used in the sterilization of medical devices [21]. These are effective against various pathogens such as bacteria, fungi, viruses [22,23,24]. CAP is therefore also used successfully against biofilms [25]. These properties are also valued in dentistry [26]. Due to its low, body-like temperature, CAP is very suitable for medical applications and has been successfully used in the treatment of skin diseases and chronic wounds for many years [27]. The anti-oncological effects of CAP have already been demonstrated in vitro in various types of cancer including glioblastoma [28], pancreatic cancer [29], melanoma [30], prostate cancer [18], head and neck cancer [31], colon cancer [32], lung cancer [33], and leukemia [34]. The in vivo anti-tumoral effects of CAP have also been demonstrated in pancreatic cancer [35], melanoma [36], ovarian cancer [37], breast cancer [38], and colon cancer [39]. Meanwhile, there are also first clinical observation case studies on the anti-tumor effects and the advantageous relief of symptoms in cancer patients due to the use of CAP [40,41,42,43]. However, this new method has mainly been used in the area of easily accessible tumors and superficial lesions, as a supplementary procedure or as a precautionary measure, especially in the palliative stage. The therapeutic induction of apoptosis is the desired effect in chemotherapy, to avoid undesired inflammation as seen with necrosis. One of the most important cellular effects of a CAP treatment also appears to be the induction of apoptotic machinery. The cell membrane is responsible for maintaining electrochemical and osmotic gradients and is therefore of crucial importance for the physiological functions of the cell [44]. However, the detailed effects of CAP therapy on the OS are not fully understood. In particular, no studies on the impact of CAP on ES cells are known so far.

## 2. Results

### 2.1. Proliferation

**Figure 1:**
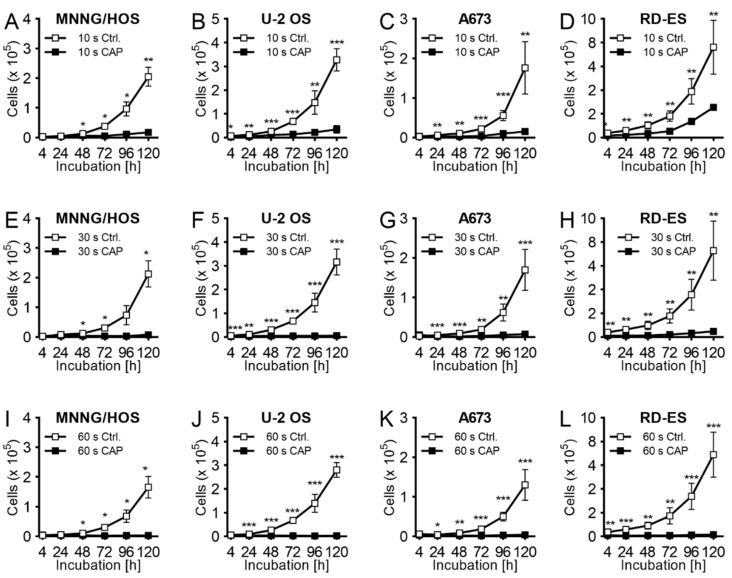
Growth inhibition effect of cold atmospheric plasma (CAP) exposure. The human OS cell lines MNNG/HOS (**A**,**E**,**I**) and U-2 OS (**B**,**F**,**J**) and ES cell lines A673 (**C**,**G**,**K**) and RD-ES (**D**,**H**,**L**) were treated for 10 s (**A**–**D**), 30 s (**E**–**H**), or 60 s (**I**–**L**) with CAP with kINPen MED. As a control group the same cell lines were treated only with carrier gas argon (CAP swiched off). The treated cells were cultivated over 120 h. The number of viable cells was counted at 4, 24, 48, 72, 96, and 120 h after exposure by using CASY cell counter and analyzer. Data show mean ± SD; Means were tested for significant differences with a paired t-test and indicated as followed: * *p* ≤ 0.05, ** *p* ≤ 0.01, *** *p* ≤ 0.001.

For the characterization of the effect of CAP treatment on bone sarcoma cells human cell lines of OS (MNNG/HOS, U-2 OS) and ES (A673, RD-ES) were used. Even a short treatment of (10 s) already showed an inhibition of cell growth. The reduction after 120 h of incubation was 92 ± 1% (*p* < 0.001 MNNG/HOS), 90 ± 2% (*p* < 0.001, U-2 OS), 90 ± 5% (*p* < 0.001, A673), and 65 ± 8% (*p* < 0.001, RD-ES) compared to cells (ctrl) treated only with argon gas (Figure 1A–D). The extension of the CAP treatment times to 30 s (Figure 1E–H) and 60 s (Figure 1I–L) caused a significant increase in the antiproliferative effect on the cancer cells. These results were observed particularly strongly for all cell lines examined after the maximum treatment time of 60 s.

**Figure 2:**
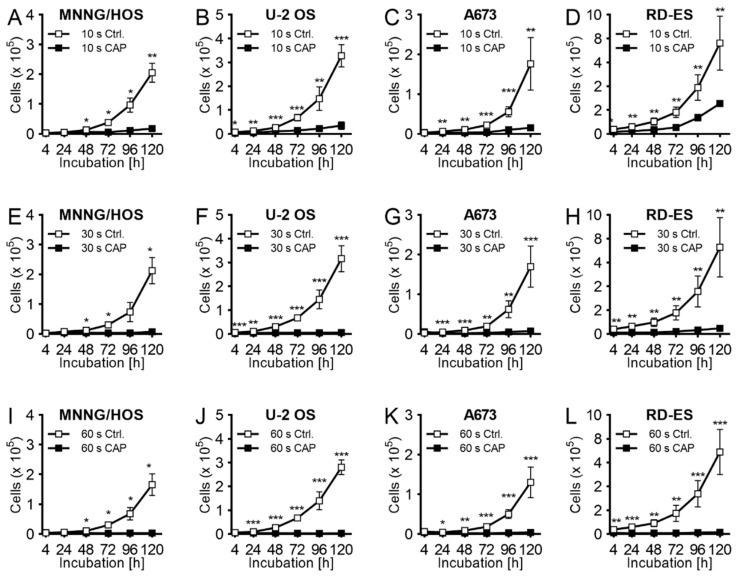
Growth inhibition effect of CAP activated media (CAM). The human OS lines MNNG/HOS (**A**,**E**,**I**) and U-2 OS (**B**,**F**,**J**), and the ES cell lines A673 (**C**,**G**,**K**) and RD-ES (**D**,**H**,**L**) were treated 24 h after seeding with CAM. For CAM treatment, cell culture media was exposed for 10 s (**A**–**D**), 30 s (**E**–**H**), or 60 s (**I**–**L**) to CAP or carrier gas argon with kINPen MED. The number of viable cells was counted at 4, 24, 48, 72, 96, and 120 h after exposure. Data show mean ± SD; Means were tested for significant differences with a paired and indicated as followed: * *p* ≤ 0.05, ** *p* ≤ 0.01, *** *p* ≤ 0.001.

The growth-inhibiting effects were also described in the so-called indirect CAP treatment, in which the cell culture medium was exposed to plasma even before the tumor cells were added. After appropriate treatment of the medium (10, 30, or 60 s) it was immediately applied to untreated bone sarcoma cells. The cells themselves were sown 24 h beforehand. As in the case of direct CAP treatment, the indirect treatment also showed a strong dependence on the duration of treatment. The antiproliferative effect after 120 h of incubation with short-term CAP-treated medium (10 s) was rather moderate in all cell lines (MNNG/HOS: 57 ± 3%, *p* = 0.002; U-2 OS: 42 ± 5%, *p* < 0.001; A673: 50 ± 23%, *p* = 0.017; RD-ES: 35 ± 4%, *p* < 0.001; Figure 2A–D). The growth inhibition after 30 s of CAP treatment of the medium was even stronger (Figure 2E–H). Ultimately, the 60 s CAP treatment of the medium achieved a similar effect to that of the direct CAP treatment of the cells (MNNG/HOS: 68% ± 8%, *p* = 0.004; U 2 OS: 69% ± 8%, *p* < 0.001; A673: 87% ± 4%, *p* < 0.001; RD-ES: 94% ± 2%, *p* < 0.001; Figure 2I–L).

### 2.2. Membrane and Cytoskeleton

**Figure 3:**
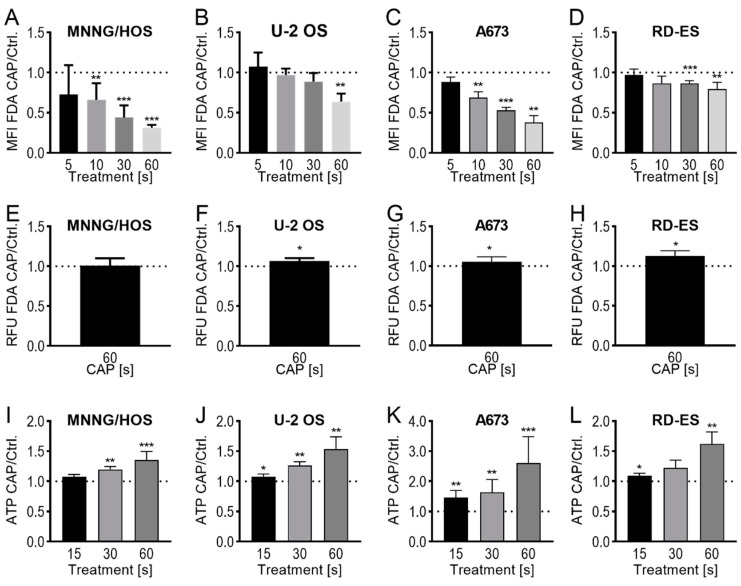
CAP treatment affects the integrity of the cell membrane. Fluorescein diacetate (FDA) uptake assay (**A**–**D**): the cell lines MNNG/HOS (**A**), U-2 OS (**B**), A673 (**C**), and RD-ES (**D**) were treated with 5, 10, 30, or 60 s of CAP or carrier gas argon, and stained with FDA-and ethidium bromide (EtBr). Afterward, the flow cytometric analysis of the viable cell was performed. For each cell line, the MFI of CAP treated cells was normalized to the MFI of argon treated cells. FDA-release assay (**E**–**H**): the cell lines MNNG/HOS (**E**), U-2 OS (**F**), A673 (**G**), and RD-ES (**H**) were incubated with FDA, washed, and treated with 60 s of CAP or carrier gas argon. After an incubation time of 20 min, the cell-free supernatant was analyzed in a fluorescence plate reader. For each cell line, the RFU of the supernatant of CAP-treated cells was normalized to the RFU of the supernatant of argon gas-treated cells. ATP-release assay (**I**–**L**): MNNG/HOS (**I**), U-2 OS (**J**), A673 (**K**), and RD-ES (**L**) were treated for 15, 30, or 60 s with CAP or carrier gas argon. After an incubation period of 3 min, the relative ATP concentrations of the cell-free supernatants were measured. The data for CAP treatment were normalized to that of the respective controls. Data show mean ± SD; significant differences were examined using a paired t-test: * *p* ≤ 0.05, ** *p* ≤ 0.01, *** *p* ≤ 0.001. MFI: mean fluorescence intensity, RFU: relative fluorescence units.

As a mixture of highly reactive species, CAP has the potential to interfere directly with the cytoplasmic membrane, as these are sensitive to physical and chemical influences. It is therefore interesting to investigate whether, under the conditions of CAP influence, translocation of various small molecules through the cytoplasmic membrane can occur. To answer this question, bone sarcoma cells were treated with CAP for 5, 10, 30 or 60 s and then incubated with FDA. When the FDA once gets into the cell, it is transformed by intracellular esterases into fluorescein. As in this form it is no longer permeable to the membrane, it accumulates in the cell. The flow cytometric analysis of the CAP treated cells thus showed a decrease in the intracellular fluorescein concentration depending on the treatment time. In comparison to cells treated only with argon gas (ctrl), fluorescein signals were detected after the maximum duration of the CAP treatment (60 s) in 31 ± 4%, (*p* < 0.001, MNNG/HOS), 63 ± 9%, (*p* = 0.002, U-2 OS), 38 ± 8%, (*p* < 0,001, A673), and 80 ± 7%, (*p* = 0.003, RD-ES) (Figure 3A–D). The expected efflux of the non-membrane permeable FDA fluorescent dye after CAP treatment was confirmed by the use of a release assay.

For this purpose, after CAP treatment, bone sarcoma cells were incubated together with FDA and after washing, the release of fluorescein in the cell culture supernatant was detected. Compared to control cells, with the exception of MNNG/HOS cells (103 ± 9%, *p* = 0.956), a modest but significant increase of the release of fluorescein was detected after CAP treatment (U-2 OS: 107 ± 4%, *p* = 0.029; A673: 106 ± 6%, *p* = 0.032; RD-ES: 113 ± 5%, *p* = 0.025; Figure 3E–H).

The ATP assay was used as another method to detect the release of small molecules after CAP treatment. This was done by measuring the ATP released from the cells into the cell culture supernatant after various CAP treatment times and after only 3 min of incubation. By extension of the treatment times (15, 30 and 60 s), the extracellular ATP concentration increased continuously and reached relative concentrations of 135 ± 12% (*p* = 0.014, MNNG/HOS), 153 ± 17% (*p* = 0.012, U-2 OS), 261 ± 76% (*p* = 0.035, A673), and 162 ± 17% (*p* = 0.008, RD-ES) of the control preparations after 60 s of CAP treatment (Figure 3I–L).

**Figure 4:**
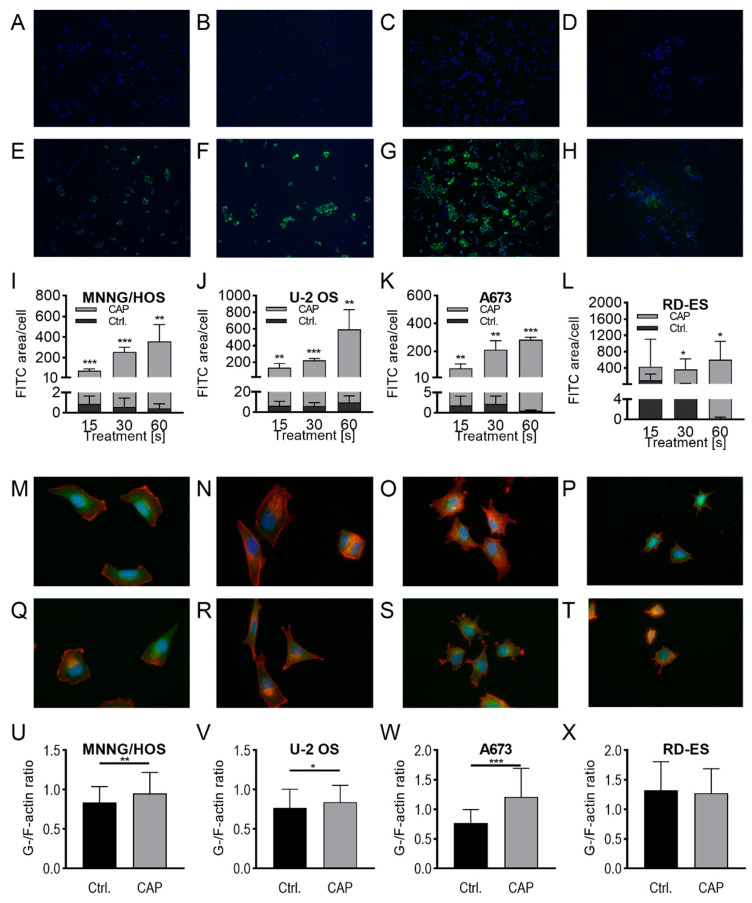
CAP treatment modulated the cytoskeleton and membrane integrity. Dextran-uptake assay (**A**–**L**): control-treated cells (**A**–**D**) and CAP-treated cells (**E**–**H**), MNNG/HOS (**A**,**E**,**I**), U-2 OS (**B**,**F**,**J**), A673 (**C**,**G**,**K**), and RD-ES (**D**,**H**,**L**) cells were seeded on cover slides and allowed to attach for 24 h. After incubation, cells were treated with 15, 30, or 60 s of CAP or carrier gas argon. Fluorescence microscopy was used to analyze of FITC dextran and DAPI. The FITC-positive areas were measured and normalized to the count of DAPI labeled cell cores. G-F-actin assay (**M**–**X**): MNNG/HOS (**M**,**Q**,**U**), U-2 OS (**N**,**R**,**V**), A673 (**O**,**S**,**W**), and RD-ES (**P**,**T**,**X**) cells were seeded on cover slides and allowed to attach for 24 h. After incubation, the cells were treated with 5 s (A673, RD-ES) or 10 s (MNNG/HOS, U-2 OS) of CAP or carrier gas argon. Cells were labeled with TRITC conjugated Phalloidin and Alexa Fluor 488 conjugated Deoxyribonuclease I, and analyzed using fluorescence microscopy. The ratio of the red (TRITC) to green (Deoxyribonuclease I) signal cell was calculated for each. Data show mean ± SD; significant differences were indicated according to a paired t-test as followed: * *p* ≤ 0.05, ** *p* ≤ 0.01, *** *p* ≤ 0.001.

Subsequently, the loss of membrane integrity was investigated using fluorescence microscopy. Fluorescein isothiocyanate (FITC) conjugated 10 kDa dextran was increasingly incorporated into the sarcoma cells after CAP treatment (Figure 4A–H). With prolonged treatment times the FITC signal per cell increased, and after 60 s of CAP treatment (Figure 4E–H) vs. the control-treated cells (Figure 4A–D). The FITC area/cell was up to several hundred times higher than in argon gas control-treated cells (MNNG/HOS: 813-fold, *p* = 0.007; U-2 OS: 60-fold, *p* = 0.005; A673: 616-fold, *p* < 0.001; RD-ES: 4627-fold, *p* = 0.049; Figure 4I–L). In RD-ES cells, the unspecific background was significantly higher in argon gas control-treated cells (Figure 4H) than in the other sarcoma cell lines. Further microscopic analyses demonstrated that the actin composition of the cytoskeleton was affected by CAP treatment, although the effects were inconsistent (Figure 4M–X). The G-/F-actin ratio was significantly increased in MNNG/HOS, U2-OS and A673 cells after 10 respectively 5 s CAP exposure (MNNG/HOS: 113%, *p* = 0.004; U-2 OS: 109%, *p* = 0.049; A673: 157%, *p* < 0.001; Figure 4U–X). In RD-ES cells, no CAP effect on the G-/F-actin ratio was demonstrated (*p* = 0.519; Figure 4X).

### 2.3. Apoptosis

**Figure 5:**
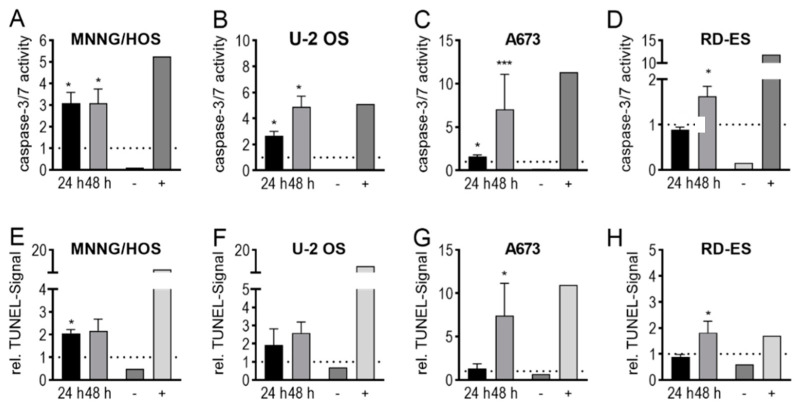
CAP treatment induced apoptosis. The human OS cell lines MNNG/HOS (**A**,**E**) and U-2 OS (**B**,**F**), and the ES cell lines A673 (**C**,**G**) and RD-ES (**D**,**H**) were treated with CAP or carrier gas argon. After 24 h and 48 h, the activity of the caspases 3 and 7 was measured (**A**–**D**), and TUNEL assays were performed (**E**–**H**). As positive control (+) was used for caspase-3/7 staurosporine, for TUNEL nuclease. Data show mean ± SD, significant differences were indicated by using a paired t-test as follows: * *p* ≤ 0.05, *** *p* ≤ 0.001.

Both changes in membrane and cytoskeleton structure as well as restrictions in cell metabolism due to energy loss can significantly impair cell homeostasis and ultimately lead to cell demise. To investigate the mechanism of cell death, the activation of the apoptosis-associated proteases caspase-3 and caspase-7 and the degradation of genomic DNA during subsequent apoptosis were tested. The caspase-3/7 assay demonstrated in both OS lines (MNNG/HOS—24 h: 308 ± 42%, *p* = 0.020, 48 h: 308 ± 54%, *p* = 0.032; U-2 OS—24 h: 266 ± 29%, *p* = 0.0148, 48 h: 489 ± 68%, *p* = 0.0149) and in an ES cell line (A673—24 h: 159 ± 18%, *p* < 0.001, 48 h: 704 ± 368%, *p* < 0.001) after 24 h as well as after 48 h a significant activation of apoptosis-specific caspases compared to control cells (Figure 5A–C). In contrast, the analysis of the RD-ES cells only revealed a tendency to increased caspase activity after 48 h (24 h: 89 ± 5%, *p* = 0.082, 48 h: 163 ± 18% *p* = 0.039; Figure 5D). These results were confirmed by using TUNEL assay detecting the subsequent DNA degradation. Such an increase after CAP treatment of osteosarcoma cells was shown after 24 h and 48 h, although without significance. (MNNG/HOS—24 h: 205 ± 12%, *p* = 0.024, 48 h: 216 ± 37% *p* = 0.106; U-2 OS—24 h: 192 ± 63%, *p* = 0.213, 48 h: 259 ± 42%, *p* = 0.112; Figure 5E–F). In Ewing’s sarcoma cell lines, however, a significantly increased degradation rate was detected only after 48 h of incubation (A673—24 h: 131 ± 48%, *p* = 0.214, 48 h: 743 ± 396%, *p* = 0.008; RD-ES - 24 h: 88 ± 7%, *p* = 0.184, 48 h: 181 ± 31%, *p* = 0.015; Figure 5G–H).

**Figure 6:**
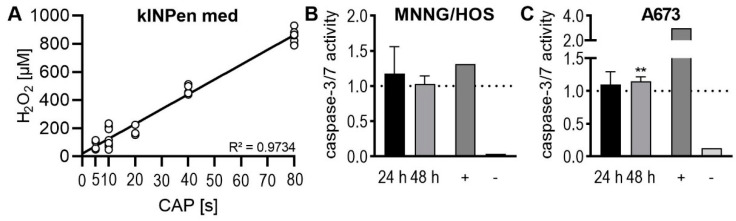
Produce of H_2_O_2_ by CAP-treatment and its influence on apoptosis. Ultrapure water was treated with CAP for 5, 10, 20, 40, and 80 s (**A**). H_2_O_2_ concentrations were determined with Ample Red Hydrogenperoxid/Peroxidase-Assay-Kit. Representative of a human OS cell line MNNG/HOS (**B**) and a human ES cell line A673 (**C**) were treated with 100 µM H_2_O_2_. After 24 h and 48 h, the activity of the caspases 3 and 7 was measured. As positive control (+) staurosporine was used, (−) are unlabeled cells (**B**, **C**). Data show mean ± SD, significant differences were indicated by using a paired t-test as follows: ** *p* ≤ 0.01.

In order to investigate the mechanism of action of the CAP, the formation of H_2_O_2_ in ultrapure water was measured. The CAP treatment of the liquid obviously creates H_2_O_2_ (Figure 6A). There is a clear linear relationship between the treatment duration and the measured H_2_O_2_ concentration (R^2^ = 0.9734). To investigate the mechanism of apoptosis proteases caspase-3 and caspase-7 were tested. The caspase-3/7 assay demonstrated in both bone sarcomas increased activity. In OS lines MNNG/HOS—24 h: 117 ± 27%, *p* = 0.485, 48 h: 103 ± 9%, *p* = 0.714 this increase was not statistically significant. In ES cell line (A673—24 h: 110 ± 13%, *p* = 0.415, 48 h: 115 ± 4%, *p* = 0.015) there was a significant activation of apoptosis-specific caspases after 48 h (Figure 6B,C).

## 3. Discussion

The therapy of skeletal sarcomas is an interdisciplinary task in which only the combination of surgical local therapy with extensive resection limits framed by systemic neoadjuvant and postoperative chemotherapy promises long-term success of the treatment. The patient’s response to postoperative chemotherapy is the decisive prognostic factor. A possible combination of this conventional approach with further innovative approaches would probably increase the chances of success significantly.

The anti-oncological effect of CAP has been demonstrated in various tumor entities such as ovarian, breast, and pancreatic cancer [29,45,46] as well as OS [45,47,48]. In OS cells, CAP treatment led to the activation of p53, to apoptosis-specific pycnotic modifications of cell morphology, and finally to the apoptotic degradation of genomic DNA [18,49,50]. The induction of apoptotic cascades and the inhibition of cell growth was independent of the CAP device used and therefore seems to represent a general CAP efficacy [47]. According to the composition of CAP, the treatment of cells leads to redox stress with specific cell responses, such as peroxiredoxin expression, which are involved in the molecular cell response leading to the induction of apoptosis [51]. Further studies on the effect of CAP treatment on the formation of soluble signal molecules (e.g., cytokines, growth factors) in OS cells demonstrated a comparatively moderate effect such as altered expression profiles of interleukins and, in particular, the suppression of the angiogenic vascular endothelial growth factor (VEGF) suggest the influence on the tumor cell’s microenvironment [52]. A possible effect of CAP treatment can therefore not only be sought directly on the tumor, but also on its microenvironment. A possible limitation of angiogenesis can influence tumor development and reduce metastasis. There is currently a large heterogeneity in the clinical applications of CAP in oncology. The type of application also varies from direct patient treatment to the development of new pharmaceutical products for antitumor therapy. For this reason, basic research and development of application protocols for each individual type of cancer are essential prior to the clinical application on patients [53]. One of the important effects of CAP, which makes the technology so desirable for intraoperative oncological applications, is its long-lasting inhibition of cell proliferation, even after a short single treatment of tumor cells. The CAP effect on OS and ES cells was profound, showing maximum growth-inhibitory effect after a treatment time of only 10 s. In RD-ES cells, a certain treatment time-dependent effect of CAP exposure was observed, as it was often described in other entities [18,54]. However, the CAP effects are not only limited to direct contact with tumor cells but are also mediated by liquid media (indirect CAP treatment) such saline solutions [55,56]. Indirect CAP treatment was also effective, although to a less pronounced amplitude as discussed previously [57,58,59]. Reactive oxygen and nitrogen species (RONS) are considered to be the main agents of anti-cancer effects after CAP treatment, including short-lived species (ONOO^−^, OH^−^, NO^−^, O_2_^−^, etc.) and long-lived species (O_3_, H_2_O_2_, NO_2_^−^, etc.) [60,61,62]. Our current experiments show that CAP treatment leads to the production of H_2_O_2_. There is a clear proportional relationship between the duration of the application and the resulting concentration of H_2_O_2_. In addition, the caspase assay performed with H_2_O_2_ show a tendency of the increased apoptotic processes in both bone sarcoma entities. The increase of intracellular RONS concentrations in cancer cells is expected to lead to the therapeutically desired effects [63]. RONS effects finally lead to an imbalance of the cellular redox system and, in addition to other factors of CAP treatment, induce cell death [56]. We have already shown the partial neutralization of CAP effects by simultaneous incubation with scavengers for OS cells [44]. Notwithstanding, indirect CAP treatment would be advantageous in oncological applications, especially for resection residues in the peripheral surgical area with metastatic potential, which would also be inactivated. The details of the redox chemistry, that are central to these mechanisms are subject of current investigation [64].

Compared to other physical therapies such as electrical, thermal, or laser-based procedures, the application of CAP lacks the induction of local tissue necrosis [65]. This is beneficial because necrosis is usually followed by inflammation, swelling, and pain [66]. Apoptosis is the regular cell death pathway induced in CAP treated tumor cells. Controlled degradation of cells and their cellular components prevents local tissue inflammation and leads to a clinical tolerability of CAP treatment. In order to demonstrate the apoptotic processes after CAP treatment, TUNEL and caspase 3/7 assays were used. Especially the effector caspases play an important role in the execution phase of apoptosis [67], in particular, caspase 3 initiates DNA fragmentation by endonucleases and the proteolytic degradation of the cytoskeleton [68]. As expected, treatment with CAP also led to the induction of apoptosis in OS and ES cells. This is certainly due to the high levels of CAP-generated reactive species, which can trigger cell cycle arrest and programmed cell death [69]. Another effect of CAP treatment was the changes in the membrane and cytoskeletal architecture, which led to cellular dysfunction. This CAP-induced damage has already been described for bacterial membranes [70,71,72,73], but the CAP effect on the eukaryotic cytoplasmic membrane is poorly explored to date. The passage of molecules with different biochemical properties and different sizes (FDA, ATP, 10 kDa dextran-FITC) through the cell membrane was shown after CAP treatment in this study using various methods. Different FDA assays were carried out to examine the effect of CAP on the cell membrane. By combining measurements of the intracellular dye concentration and the cell-free supernatant concentration, some FDA-related restrictions are avoided. FDA is hydrolyzed intracellularly, which after excitation leads to the fluorescence of the dye and can no longer leave the cell if the cell membrane is intact. A reduction of the intracellular dye concentration therefore suggests membrane damage [74]. However, the measurement results can be influenced by reduced absorption of FDA into the cell or reduced hydrolysis by influencing the esterases after CAP treatment. This limitation is compensated by the use of the FDA release assay, since in this assay the cells are first loaded with FDA and then CAP treatment is performed. Thus dye absorption and dye hydrolysis are independent of CAP treatment. From a critical point of view, an increase of fluorescein concentration in the cell-free supernatant can also occur due to a possible CAP-induced lysis of single cells. This misrepresentation of the results does not occur in the dye uptake assay, because only living cells will be considered here. Only by combination of both methods it is possible to interpret the findings as an indication of damage to membrane integrity by CAP treatment. In addition, the results of the FDA release assays were confirmed by the ATP release assay [75]. ATP release measurement is an established method for the detection of membrane defects in non-tumor and tumor cells [76,77,78] and has been demonstrated before after CAP treatment [79,80,81,82,83]. The CAP-induced loss of ATP leads to disturbances in cellular metabolism and might be part of the antiproliferative effect of CAP treatment. However, extracellular ATP can also serve for activation of macrophages in an immunological context [84]. The underlying molecular mechanism of the molecule transfer through the cytoplasmic membrane is unclear and subject of current research. Since the different test molecules FDA, ATP, and 10 kDa dextran-FITC passed the cytoplasmic membrane after CAP treatment, a rather unspecific mechanism can be assumed. The highly reactive properties of the CAP composition rather indicate direct physicochemical modifications of membrane components, e.g., lipid and protein oxidation or activation of membrane-bound cellular redox mechanisms [14,85,86,87].

These reactive properties were also observed in the analysis of the actin components of the cytoskeleton. With the exception of the ES cells RD-ES, all sarcoma cells showed a significant increase in the ratio of G-actin to F-actin. The depolymerization of the actin filaments may result from a direct physicochemical interaction of the CAP components with the actin. However, it is also conceivable that redox-active components of CAP cause corresponding signal cascades, which ultimately lead to the activation of actin-binding proteins and subsequent actin remodeling [88]. Currently, there is very little data on the effects of CAP treatment on the architecture and function of the eukaryotic cytoskeleton. In addition, the limited number of studies is ambiguous in terms of mechanisms [89,90,91,92]. If components of CAP lead to modifications of the cytoskeleton, however, it can be assumed that, in addition to changes in the cytoplasmic membrane, modifications of the cytoskeleton structure affect cellular functionality and physiology. Similar to the inactivation of bacteria [87], CAP treatment may lead to permeabilization of the cell surface, resulting in loss of membrane integrity and leakage of intracellular components.

It has to be discussed whether the effect of CAP treatment is specific for malignant cells or whether adjacent non-malignant cells are also affected. This is particularly relevant for clinical application because, depending on the entity, non-malignant tissue sections would most likely also come into contact with CAP as a result of the treatment. Canal et al. confirmed the selectivity of CAP treatment on osteosarcoma cell lines in contrast to normal non-malignant cells (osteoblasts and human mesenchymal stem cells) [93]. However, the selective anti-cancer potential of CAP can also be attributed to the combined effect of several cellular factors, such as, for example, the increased expression of membrane channels or the reduced expression of specific antioxidative enzymes in cancer cells [58], and due to the higher sensibility of cancer cells to oxidative stress as a result of their high metabolic rate [56]. Further experiments, especially in vivo models, will be necessary in the future to answer the possible clinical applications and side effects of CAP treatment in bone sarcomas.

## 4. Materials and Methods

### 4.1. Cell Culture

Human OS cell lines MNNG/HOS and U-2 OS (Cell Lines Service, Eppelheim, Germany) as well as ES cell lines A673 (American Type Culture Collection, Manassas, VA, USA) and RD-ES (DSMZ-Deutsche Sammlung von Mikroorganismen und Zellkulturen, Braunschweig, Germany) were propagated in Dulbecco’s modified Eagle’s medium (DMEM) containing 1.0 g/L glucose, 10% fetal bovine serum, 1 mM sodium pyruvate, and 1% penicillin/streptomycin (MNNG/HOS, U-2 OS, A673) or Roswell Park Memorial Institute (RPMI) 1640 containing 10% fetal bovine serum and 1% penicillin/streptomycin (RD-ES) (all PAN Biotech, Aidenbach, Germany) in a humidified atmosphere at 5% CO_2_ and 37 °C.

### 4.2. CAP-Treatment

**Figure 7:**
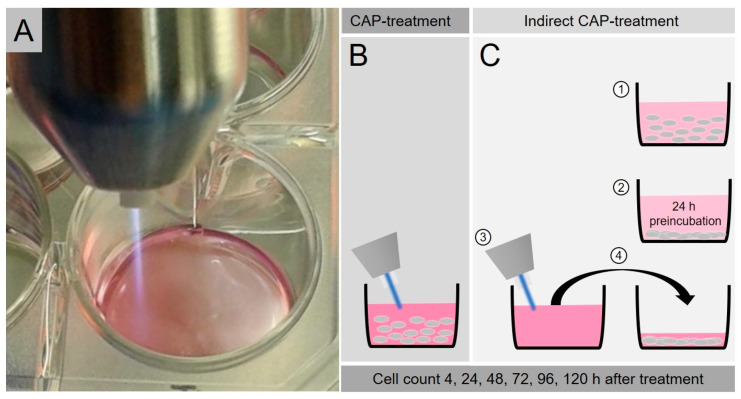
CAP Treatment. Manual Treatment Procedure of CAP (**A**). The direct CAP exposure of the cells in suspension (**B**). The indirect CAP treatment (**C**): The cells are not exposed to the CAP, but treated with CAP-activated medium.

The cold atmospheric pressure plasma jet kINPen MED (neoplas tools, Greifswald, Germany) was used for CAP treatment. As a carrier gas, argon was used (Alphagaz 1 AIR LIQUIDE Deutschland, Düsseldorf, Germany) with a gas flow of 3 L/min. The device has a voltage supply of 65 V DC with a frequency of 1.1 MHz. The treatment was carried out by manually guiding the pen over the suspension at a standard distance so that the plasma flame reached the surface (Figure 7A). In order to ensure an even exposure of the entire volume of the well, the pen was meandered over the treated surface.

As a control, an analogous treatment was carried out only with argon gas without a power supply and thus without the formation of CAP.

The principles of direct and indirect CAP treatment are shown schematically in Figure 7B,C. In the case of direct CAP treatment, the cells are suspended in the medium and directly exposed to CAP exposure there (Figure 7B). In the indirect CAP treatment (Figure 7C), on the other hand, the cells are not exposed to the CAP, but rather cultivated with CAP-activated medium (see Section 4.3 and Section 4.4. of the proliferation assays).

### 4.3. Proliferation Assay after CAP-Exposure

Cell growth was determined after 4, 24, 48, 72, 96, and 120 h using a CASY cell counter and analyzer model TT (Roche Applied Science, Mannheim, Germany) with a 150 μm capillary. For this purpose, 1 × 10^4^ (U-2 OS, MNNG-HOS, A673) and 2.5 × 10^4^ cells were suspended in 200 μL culture medium and treated with CAP or carrier gas argon (control group) for 10, 30, or 60 s. After treatment, 800 μL of fresh medium was added, and the cells were incubated in a humidified atmosphere at 5% CO_2_ and 37 °C over 4, 24, 48, 72, 96, and 120 h. Cell counting was done by suspending cells via trypsin/EDTA treatment and diluting 100 μL cell suspension in 10,000 μL CASYton (Roche Applied Science). Measurement was performed three times with 400 μL each of this dilution and was performed in triplicates. To discriminate live cells from cell debris and dead cells, gates of 7.20 μm/13.95 μm (U-2 OS), 7.20 μm/14.85 μm (MMNG-HOS), 5.63 µm/9.5 µm (A673), and 7.45 µm/10.2 (RD-ES) were used.

### 4.4. Proliferation Assay after Indirect CAP-Exposure

In total, 0.5 × 10^4^ (U-2 OS, MNNG-HOS, and A673) and 1.25 × 10^4^ (RD-ES) cells were pre-incubated over 24 h in a humidified atmosphere at 5% CO_2_ and 37 °C. The cell culture medium was removed, and cells were treated with 200 µL CAP-activated medium. For this purpose, 200 µL medium were treated with 10, 30, or 60 s of CAP or carrier gas argon in another 24-well cell culture plate. Cells counts were performed at 4, 24, 48, 72, 96, and 120 h after the indirect CAP-exposure as described in proliferation assay after direct CAP treatment.

### 4.5. FDA-Uptake Assay

**Figure 8:**
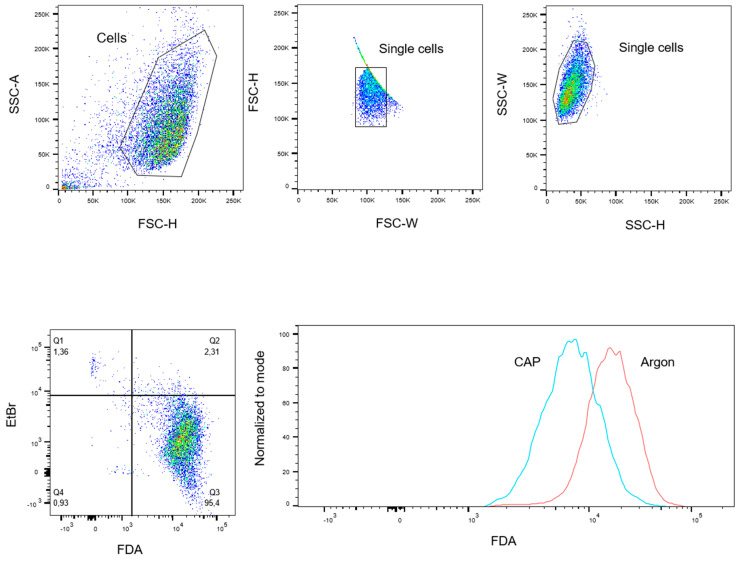
Gating Strategy. Fluorescein diacetate (FDA) content per cell was analyzed by flow cytometry. Debris and doublets were excluded by forward and side-scatter characteristics. Living cells were defined as ethidium bromide (EtBr)-negative and FDA-positive events. For analysis of data, the mean fluorescence intensity (MFI) of FDA was compared. SSC-A: side-scatter area, SSC-H: side-scatter height, FSC-W: forward-scatter width, FSC-H: forward-scatter height.

Cells were harvested and diluted to 1 × 10^6^ cells per mL with measuring buffer (Dulbecco’s Phosphate Buffered Saline (DPBS) with 10% FCS *v*/*v*). The cell suspension was stored on ice until use. A dye solution containing 30 μg/mL ethidium bromide (Carl Roth, Karlsruhe, Germany) and 5 μg/mL FDA (Sigma-Aldrich, St. Louis, MI, USA) in DPBS was prepared. Two-hundred microliters of CAP or untreated cells were added to 200 μL of Ethidium bromide/FDA dye solution and incubated for 15 min in the dark on ice. After centrifugation (5 min at 300× *g* and 4 °C), labeled cells were resuspended in PBS and analyzed using a FACSCantoTM flow cytometer with FACSDivaTM 6.0 Software (both BD Biosciences, Heidelberg, Germany) and evaluated with FlowJo Software Version 10 (Tree Star Inc., Ashland, TN, USA). Ethidium bromide-positive cells were discriminated as dead cells. Figure 8 shows a representative example of the gating strategy used. Data of CAP treated cells were normalized to the control cells. Color compensation was performed by the control and analysis software. As a reference, living and dead cells were measured, with both dyes separated and then measured together.

### 4.6. FDA-Release Assay

An acetone stock solution with 10 mg/mL FDA was prepared for the detection of the release of FDA from FDA loaded cells, and diluted to a concentration of 5 μL/mL with PBS for the measurement. The cells were loaded with FDA for 30 min in the dark on ice. Excess FDA was removed by washing three times (3 min at 150× *g* and 4 °C) and resuspended in PBS. Two-hundred microliters of suspension were treated with CAP or carrier gas argon for 60 s and incubated on ice for 20 min in the dark. Subsequently, the cells were sedimented (3 min at 150× *g* and 4 °C) and 100 μL of the cell-free supernatant were analyzed in an Infinite m200 PRO multimode plate reader (Tecan, Männedorf, Switzerland) with an excitation wavelength of 300 nm and an emission wavelength of 520 nm. The CAP treatment measurements were normalized to the control measurements.

### 4.7. ATP-Release Assay

Cells were harvested and adjusted to 10^6^ cells/mL with PBS. Two-hundred microliters of cell suspension were treated with CAP or carrier gas argon (control group) for 15, 30, and 60 s. After treatment, cells were incubated over 3 min and sedimented (5 min at 300× *g* and 4°C). Relative ATP concentrations of cell-free supernatants were measured using the CellTiter-Glo 2.0 Reagent (Promega GmbH, Walldorf, Germany) in an Infinite m200 PRO multimode plate reader (Tecan, Männedorf, Switzerland). The CAP treatment measurements were normalized to the control measurements.

### 4.8. Dextran-Uptake Assay

Cells were seeded on cover slides and incubated for 24 h (37 °C, 5% CO_2_, humidified atmosphere). After incubation, cells were washed in PBS and treated for 15, 30, and 60 s with CAP or carrier gas argon. Cells were incubated with a staining solution (150 µg/mL FITC-Dextran (average molecular weight 10,000) (Sigma-Aldrich, St. Louis, MI, USA) and 0.5 µg/mL DAPI (Thermo Fisher Scientific, Waltham, MA, USA) in PBS. After staining, cells were washed three times with PBS. Fluorescence of FITC and DAPI were recorded with a BZ-9000 microscope and analyzed with BZ-II Analyzer (Keyence, Neu-Isenburg, Germany). The FITC-positive areas were measured and normalized to the count of DAPI labeled cell nuclei.

### 4.9. G-/F-Actin Assay

Cells were seeded on cover slides and incubated over 24 h (37 °C, 5% CO_2_, humidified atmosphere). The culture medium was changed, and cells were treated for 5 s (A673, RD-ES) or 10 s (MNNG/HOS, U-2 OS) with CAP or carrier gas argon. After an incubation period of 4 h, the cells were washed with PBS and fixed with 1% paraformaldehyde (Carl Roth, Karlsruhe, Germany). Cells were permeabilized with Triton-X100 (0.3%; Carl Roth, Karlsruhe, Germany). Cells were stained for 20 min with Rhodamine conjugated Phalloidin (0.022 µM) (Thermo Fisher Scientific, Waltham, MA, USA) and Alexa Fluor 488 conjugated Deoxyribonuclease I (0.3 µM) (Thermo Fisher Scientific, Waltham, MA, USA) in the dark. DAPI (1.43 µM) (Thermo Fisher Scientific, Waltham, MA, USA) was added for the last 3 min of incubation. The fluorescence was recorded with BZ-9000 microscope and analyzed with BZ-II Analyzer software (KEYENCE, Neu-Isenburg, Germany). The ratio of the red to the green signal of each cell was calculated.

### 4.10. Caspase Assay

After CAP treatment, the activities of Caspase-3 and Caspase-7 were measured using a specific substrate peptide coupled with a fluorescent dye (CellEvent Caspase-3/7 Green Detection Reagent; Thermo Fisher Scientific, Waltham, MA, USA) according to the manufacturer’s protocol. Cells were incubated with CellEventTM Caspase-3/7 Green Detection Reagent at 37 °C for 45 min as a control, apoptosis was induced by the addition of staurosporine (100 μM in cell culture medium; Carl Roth, Karlsruhe, Germany). Cells were incubated for 45 min at 37 °C. Subsequently, the fluorescent dye was excited at 495 nm, and the emission was measured at 535 nm. Data were assessed using an Infinite 200 PRO multimode plate reader (Tecan, Männedorf, Switzerland).

The caspase measurement was carried out in the same way after the addition of H_2_O_2_ to the cells.

### 4.11. TUNEL Assay

A total of 2.5 × 10^4^ (24 h) and 5.0 × 10^4^ (48 h) U2-OS, MNNH/HOS, and RD-ES cells, respectively, and 1.0 × 10^4^ (24 h) and 2.0 × 10^4^ A673- cells, respectively, were suspended in 200 μL culture medium). CAP treatment was performed according to the manufacturer’s protocol. After incubation for 24 and 48 h, adherent cells were detached with 0.1% trypsin/0.04% EDTA. TUNEL analysis was performed using the HT TiterTACS Assay kit (Trevigen, Gaithersburg, MD, USA) according to the manufacturer’s protocol. As a positive control, cells were nuclease treated according to the supplier’s instruction. The incubation of the cells was at 37 °C for 45 min. Data were acquired using an Infinite 200 PRO multimode plate reader (Tecan, Männedorf, Switzerland).

### 4.12. Hydrogenperoxid Assay

The formation of H_2_O_2_ after CAP treatment was verified using the Molecular Probes Ample Red Hydrogenperoxid/Peroxidase-Assay-Kit (Thermo Fisher Scientific, Waltham, MA, USA). For this purpose, 200 µL of ultrapure water were treated with CAP for 5, 10, 20, 40 and 80 s. The treatment followed analogous to the treatment in the other experiments. CAP-treated water was diluted 1:100. A standard curve was created from 0–20 µM. The Assay was carried out according to the manufacturer’s instructions.

### 4.13. Data Analysis

For data analysis and visualization, Microsoft Excel Version 1903 (Microsoft Corp., Redmond, WA, USA) and GraphPad Prism Version 7.04 (GraphPad Software Inc., La Jolla, CA, USA) were used. Results of *p* ≤ 0.05 of at least three replicates were considered significant and data are given as the mean ± SD. As CAP treated and control cells were harvested from the same cell culture flask, data were examined for significant differences with the paired t-test. The normal distribution of differences between groups was checked with the Shapiro-Wilk normality test.

## 5. Conclusions

Our data suggest that physical plasma therapy of aggressive bone sarcomas (osteosarcoma, Ewing’s sarcoma) represents a promising extension of the existing therapeutic spectrum. Short treatment times already inhibited cell growth significantly by damaging the cell membranes and initiating programmed cell death. Subsequent research will need to explore the therapeutic implications of this novel type of treatment in more advanced disease models.
